# Experimentally Induced Repeated Anhydrobiosis in the Eutardigrade *Richtersius coronifer*

**DOI:** 10.1371/journal.pone.0164062

**Published:** 2016-11-09

**Authors:** Michaela Czernekova, K. Ingemar Jönsson

**Affiliations:** 1 School of Education and Environment, Kristianstad University, Kristianstad, Sweden; 2 Institute of Physiology, Academy of Sciences of the Czech Republic, Prague, Czech Republic; 3 Faculty of Medicine, Charles University, Prague, Czech Republic; University of North Carolina at Chapel Hill, UNITED STATES

## Abstract

Tardigrades represent one of the main animal groups with anhydrobiotic capacity at any stage of their life cycle. The ability of tardigrades to survive repeated cycles of anhydrobiosis has rarely been studied but is of interest to understand the factors constraining anhydrobiotic survival. The main objective of this study was to investigate the patterns of survival of the eutardigrade *Richtersius coronifer* under repeated cycles of desiccation, and the potential effect of repeated desiccation on size, shape and number of storage cells. We also analyzed potential change in body size, gut content and frequency of mitotic storage cells. Specimens were kept under non-cultured conditions and desiccated under controlled relative humidity. After each desiccation cycle 10 specimens were selected for analysis of morphometric characteristics and mitosis. The study demonstrates that tardigrades may survive up to 6 repeated desiccations, with declining survival rates with increased number of desiccations. We found a significantly higher proportion of animals that were unable to contract properly into a tun stage during the desiccation process at the 5^th^ and 6^th^ desiccations. Also total number of storage cells declined at the 5^th^ and 6^th^ desiccations, while no effect on storage cell size was observed. The frequency of mitotic storage cells tended to decline with higher number of desiccation cycles. Our study shows that the number of consecutive cycles of anhydrobiosis that *R*. *coronifer* may undergo is limited, with increased inability for tun formation and energetic constraints as possible causal factors.

## Introduction

Water availability is one of the most important ecological factors and evolutionary pressures on terrestrial life. Despite the fact that water is crucial for all life forms, numerous organisms (including prokaryotes, protozoa, fungi, plants and animals) survive temporary drying to equilibrium with the air humidity by entering a highly stable and reversible state called anhydrobiosis, a special form of the ametabolic life state known as cryptobiosis [1,2,3,4,5]. During anhydrobiosis the organism loses most of its water by evaporation and has to protect cell structures from damage caused by water loss [6]. The biochemical and physiological nature of such protectant systems in anhydrobiotic organisms are not well understood, but are of considerable interest both from a general biological perspective and from the applied sciences where dry biological systems play an important role (e.g., medicine and food storage) [7,8].

Among animals, tardigrades represent one of the main groups in which a capacity for anhydrobiosis is widespread. Tardigrades are microscopic aquatic animals found in a variety of habitats worldwide [9], and they are particularly common in semi-terrestrial microhabitats such as mosses, lichens and leaf litter. In these environments they are exposed to periods of desiccation that varies in frequency and length, and the anhydrobiotic capacity of semi-terrestrial tardigrades is an evolutionary adaptation to survive under such conditions. Their ability to enter anhydrobiosis is well documented [5,10,11], and the anhydrobiotic state may be entered recurrently and at any stage of their life cycle (so-called “holo-anhydrobiosis”; [12]).

The state of anhydrobiosis, characterized as an ametabolic condition, is not connected with any energy consumption, and this explains why tardigrades may stay in this state for many years, even decades, and still be able to revive [13,14]. However, the entrance into and exit of anhydrobiosis relies on physiological processes that are likely to be energetically costly, and evidence of energy-depletion in storage cells (coelomocytes with circulatory and energy storage functions) of tardigrades over a single cycle of anhydrobiosis has been reported [15,16]. This suggests that multiple cycles of anhydrobiosis may eventually deplete the energy stores of the animal and represent a potential constraint on how many times in a row a tardigrade may successfully enter anhydrobiosis, given that energy stores cannot be replenished by feeding. Apart from energy depletion, desiccation may also give rise to damage to cell components, including DNA [17], and multiple anhydrobiotic periods may therefore also challenge the maintenance of cell structure integrity.

Very few previous studies have evaluated how many consecutive periods of anhydrobiosis that tardigrades are able to survive. According to Baumann [18], Lance [19] was the first to examine repeated desiccation in tardigrades, reporting survival of 8–14 desiccations, but only three eutardigrade specimens were used. Baumann [18] reported the first and so far the only more extensive study on this subject, including 15 animals of the genus *Macrobiotus* (species not given). The results showed that single specimens were able to survive up to 9 repeated desiccations, but already after 5 desiccations about 50% of the animals had died. The only other study where tardigrades have been exposed to several sequential cycles of anhydrobiosis is that by Hengherr et al. [20], in which specimens of the eutardigrade *Milnesium tardigradum* were repeatedly desiccated with intermediate 7-day periods of hydration under cultured conditions allowing feeding. The study found no decline in anhydrobiotic performance over 9 consecutive desiccations.

The purpose of the current study was to investigate the patterns of survival in a tardigrade under repeated cycles of desiccation/rehydration and the potential effect of repeated desiccation on size, shape and number of storage cells. We also analyzed if body size, gut content and frequency of mitosis in storage cells change over the course of repeated desiccation.

## Materials and Methods

### Study organism

In our desiccation experiment we used medium sized (average body length of 653 μm, n = 80) specimens of the eutardigrade *Richtersius coronifer* (Richters, 1903). The population consists almost exclusively of females [21]. No culturing method has been developed for this species, and specimens were extracted from a natural moss-living population in Alvar habitat of the island Öland (see [22] for description of the Alvar area). The study did not involve endangered or protected species, and moss samples were not collected within an area where permission was required. Moss (*Orthotrichum cupulatum*) containing tardigrades were collected dry and stored at room temperature for 3 days until use. Moss cushions were hydrated for about 2 hours in tap water and within the following 3 hours 400 active specimens were extracted with sieves (mesh size 250 and 40 μm) under running tap water. Extracted animals were washed thoroughly with distilled water to remove adherent particles.

### Anhydrobiotic induction and recovery

The general procedure of our study consisted of repeated 24 hour periods of desiccation at 95% relative humidity (RH) followed by 5 hours of rehydration. These times were assumed to provide enough time for the animal to enter anhydrobiosis and to rehydrate and adjust physiologically for the hydrated state, respectively. After each desiccation cycle, 10 revived specimens were selected for analysis of mitosis in storage cells and morphometric characteristics, and the remaining animals that were alive were mixed and prepared for a new period of desiccation. This procedure was repeated until there were too few alive animals to continue.

At the start of the experiment, 400 randomly chosen specimens were put individually on ten replicate filter papers (5 x 2 cm) in petri dishes and transferred into a desiccator with a saturated potassium nitrate (KNO3) salt solution providing a relative humidity of 95%. The 24h desiccation allowed the animals to enter anhydrobiosis and equilibrate with the surrounding humidity condition. Since the total number of animals available for each new desiccation cycle steadily declined (due to removal of specimens for analysis + mortality of some specimens at each cycle), the mean number of animals in each replicate was a decreasing function of the number of desiccation cycles (1^st^ cycle = 40 specimens/replicate, 2^nd^ = 38.4, 3^rd^ = 31.5, 4^th^ = 22.9, 5^th^ = 14.9, 6^th^ = 3.5). In all desiccation cycles the number of specimens in the 10 replicates differed by maximum one animal.

Before each rehydration all specimens were examined under stereomicroscope and the number of animals in tun stage (S1A Fig), semi-tun stage (body not fully contracted, S1B Fig) and extended (non-tun) stage (S1C Fig) in each sample were recorded. Gut content was also recorded and specimens were classified as having gut content (dark gut) or no gut content (yellow gut). Twenty hydrated animals not included in the desiccation experiment were initially selected and used as controls for comparison with the repeatedly dehydrated animals.

Survival of the rehydrated animals was evaluated after 3 and 5 hours, and the latter estimate was used for most animals in the survival analysis. However, in the survival estimate we also included the 10 specimens selected for mitosis and morphometric analysis, and these were collected at the 3 hour survival estimate. All specimens alive at the 3 hour evaluation were also alive after 5 hours. No specimens inactive during all 5 hours of rehydration recovered after this time (dead specimens were examined for 24 hours). Animals were assumed to be dead if there was no visible body movement. After each rehydration period, alive specimens were put together in a single dish and transferred individually with an Irwin loop to 10 new filter papers, followed by a new period of desiccation. For each replicate sample within a desiccation-rehydration cycle the proportion of surviving animals was calculated and used in the analysis.

### Initial observations and preparation for analysis

The 10 animals randomly selected after each desiccation/rehydration cycle were transferred in a drop of water to an object slide, covered with a cover slip, and observed under light microscope (Olympus BX60) fitted with a digital camera (INFINITY 1, Lumenera Corp.). Images of the whole animal, buccal tube and storage cells were taken for later measurements using the image analysis software (INFINITY ANALYZE 6.0, Lumenera Corp.). The time for collection of all images for a single specimen was about 10 minutes. Images for measurement of buccal tube and storage cells were taken at 40X magnification.

After initial observation specimens were transferred into Carnoys´ solution for 3 hours and stained *in toto* in a drop of acetic-lactic orcein according to the method used by Rebecchi [23]. Slides were examined the following day under the light microscope.

### Analysis of morphometric characteristics and mitosis

Measured morphometric variables were body length, buccal tube length, gut content, size and shape of storage cells and presence of mitotic storage cells. Measurement of total body length was taken from anterior to posterior part of the body excluding the fourth pair of legs, and buccal tube length was measured according to the description by Pilato [24]. The measurements of body size and buccal tube length were significantly correlated (Pearson correlation; r = 0.27, N = 62, P = 0.036), and in order to include also specimens that were in a simplex stage without buccal tube (about 22% of the data) we only used body length in our analyses. Gut content was analyzed at 20X magnification and classified as empty, medium (part of gut filled with brownish coloration material) or full (gut filled with black material). Note that these analyses of gut content represent a different estimate than that made on all animals prior to rehydration (dark gut/yellow gut).

Storage cell shape and size were estimated from 20 randomly selected cells per individual (and from different parts of the bodies). Cell size was calculated as the mean of two diagonal right angle diameter measurements for each cell. From the diameter measurement cell volume was calculated (assuming a spherical shape) and used in the analysis. Shape of the cells was characterized for each examined individual as regular (spherical) or irregular (crescent or borders not straight). There were no specimens with mixed cell shapes; all cells in an animal were either regular or irregular.

In order to calculate the mitotic index (proportion of all storage cells that were in mitosis) at the individual level, the total number of clearly recognizable storage cells was estimated manually at 100X magnification with phase contrast and oil, using image analysis software (see above). Smashed cells, without clear borders, were not counted. Mitotic storage cells (S2 Fig) were identified from the presence of condensed chromosomes.

In total 420 specimens were used in the experiment, 400 specimens in the repeated desiccation sequence and 20 as controls. Mitosis and morphometric analyses were performed on 80 specimens.

### Statistical analyses

Statistical effects on survival of repeated desiccations were tested using ANOVA and linear regression analysis (IBM SPSS Statistics v.23). Proportion data were arcsin-transformed before statistical analyses. In tests where parametric assumptions were not met, non-parametric statistics were used. Associations between mitosis occurrence and desiccation cycles/phenotypic traits were analyzed with logistic regression. Statistical tests were considered significant when P < 0.05, and marginally significant when P = 0.05.

## Results

### Survival rates after repeated desiccations

**Fig 1:**
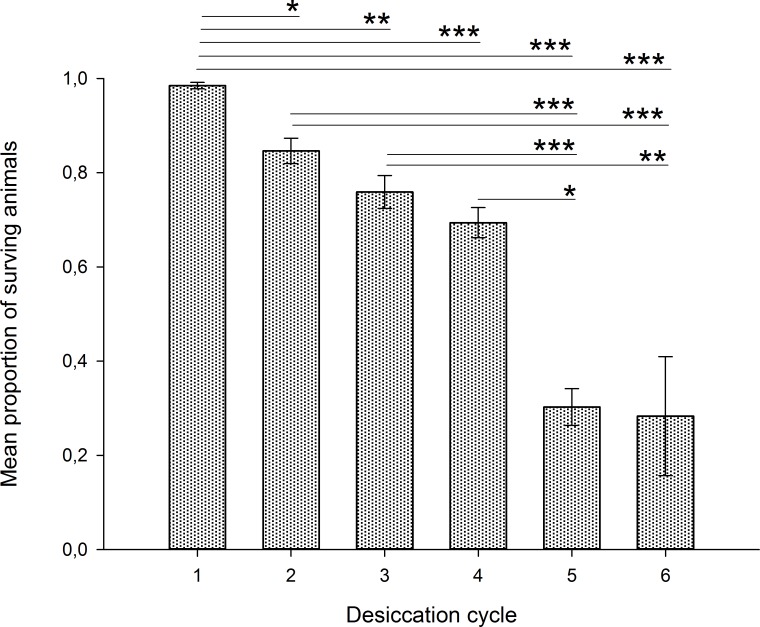
Mean survival rate after different number of repeated desiccations. Error bars indicate standard error of the mean. Significant differences at P<0.05 (*), P<0.01 (**) and P<0.001 (***) are indicated.

Survival rates differed significantly among the repeated desiccations (F_5,54_ = 20.96, P < 0.001; Fig 1 and Table A in S1 File) and the trend was a clear and significant decline in survival rate with increased number of desiccation cycles (linear regression; F_1,58_ = 87.62, r = –0.78, P < 0.001). The highest survival rate was recorded after the first desiccation (98.5%) and the lowest non-zero survival rate was recorded after the sixth desiccation (28.6%). After the 6^th^ desiccation only 10 specimens were alive and all of these were used for morphometric and mitosis analyses.

### Associations between repeated desiccations and phenotypic traits based on examination of all specimens after each desiccation cycle

**Fig 2:**
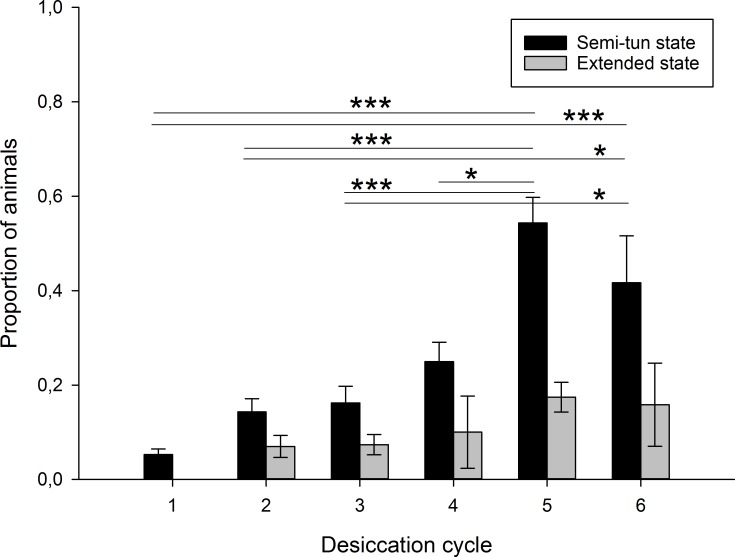
Mean proportion of animals that were in semi-tun and extended states after each respective desiccation cycle. Error bars indicate standard error of the mean. Horizontal bars indicating significances refer to the semi-tun data. Significance levels of P<0.05 (*) and P<0.001 (***) are indicated.

**Fig 3:**
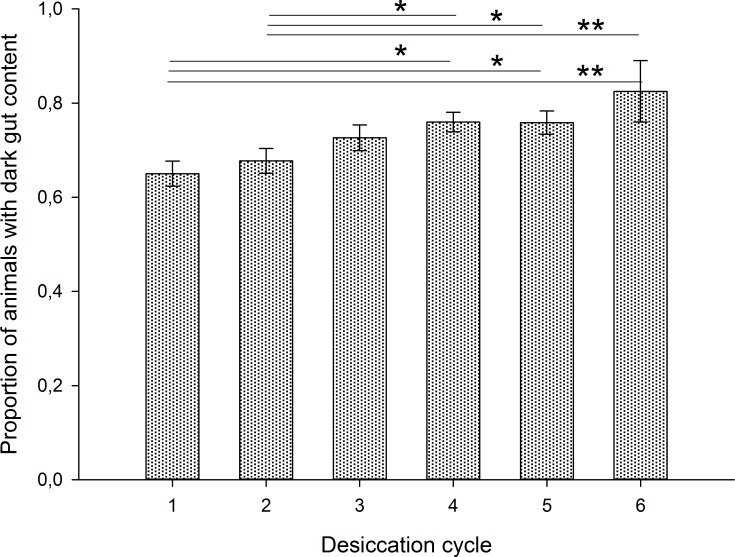
Mean proportion of animals with dark gut content after each respective desiccation cycle. Error bars indicate standard error of the mean. Significant differences at P<0.05 (*) and P<0.01 (**) are indicated.

The proportion of animals that were in a semi-tun stage after the 24h desiccation differed significantly among desiccation cycle groups (F_5,54_ = 8.96, P = 0.000), with desiccation groups 5 and 6 having significantly more semi-tuns than most of the other groups (Fig 2 and Table A in S1 File). A similar pattern was observed for animals that were in an extended state after desiccation but within-group variation was high and there were no significant differences among desiccation groups (F_5,54_ = 1.57, P = 0.19; Fig 2 and Table A in S1 File). The proportion of animals with dark gut content after desiccation differed significantly among desiccation cycle groups (Kruskal-Wallis; χ^2^ = 15.0, df = 5, P = 0.010; Fig 3 and Table A in S1 File), with the 1^st^ and 2^nd^ desiccation group having a significantly lower proportion of specimens with dark guts compared to groups 4–6.

### Associations between repeated desiccations and phenotypic traits based on 10 selected specimens after each desiccation cycle

**Fig 4:**
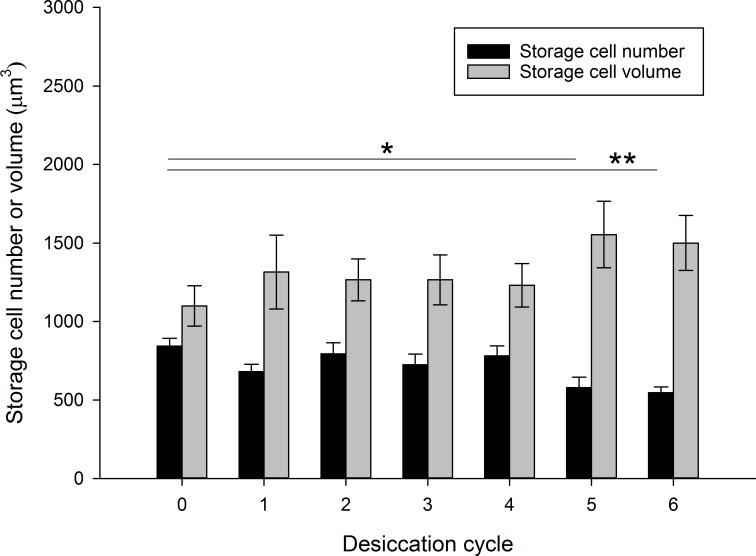
Mean number of storage cells and storage cell volume (μm^3^) in tardigrades exposed to different number of desiccation cycles. Error bars indicate standard error of the mean. Horizontal bars indicating significances refer to the storage cell number data. Significant differences at P<0.05 (*) and P<0.01 (**) are indicated.

There was an overall significant difference in the mean number of storage cells among desiccation cycle groups, including also the control group (F_6,72_ = 3.91, P = 0.002; Fig 4 and Table B in S1 File). This difference was exclusively due to significantly lower number of storage cells in animals exposed to five (P = 0.015) and six (P = 0.004) desiccation cycles, compared to the control animals. No significant differences were found among desiccation cycle groups (including controls) with respect to body length (F_6,73_ = 2.07, P = 0.067; Table B in S1 File) or storage cell volume (F_6,69_ = 0.99, P = 0.44; Fig 4 and Table B in S1 File). The storage cells had regular shape in almost all of the specimens, and only one control specimen and two specimens of the first desiccation group were characterized as having irregular cells. The proportion of animals in different gut content categories did not differ significantly among desiccation cycle groups (χ^2^ = 18.4, df = 12, P = 0.10), and there was no trend in gut content in relation to number of desiccation cycles (Spearman´s rank Analysis; r_s_ = 0.10, N = 80, P = 0.37).

### Frequency of mitosis in storage cells

Storage cells undergoing mitosis were found in 17.5% (14/80) of all examined specimens. The average percentage of mitotic storage cells among all cells (“mitotic index”) was 0.28% (SD = 1.25, n = 79) when all analyzed animals (also zero-values) were included, and 1.60% (SD = 2.65, 0.11–10.19%, n = 14) when only animals where mitosis was found were included. The proportion of animals with mitotic storage cells in each desiccation cycle varied between 0 and 0.4 (Table 1). Occurrence of mitotic cells in an individual specimen was not related to storage cell volume (Wald = 0.39, df = 1, P = 0.53), gut content (Wald = 3.02, df = 2, P = 0.22), or body length (Wald = 0.040, df = 1, P = 0.84), but was significantly negatively associated with number of desiccation cycles (ß = –0.32, Wald = 3.89, df = 1, P = 0.049) and positively associated with storage cell number (ß = 0.003, Wald = 4.064, df = 1, P = 0.044). Animals with no mitotic cells had on average 699 (SD = 221) storage cells, while animals with mitotic cells had 832 (SD = 155) storage cells. There was a significant negative correlation between the mitotic index for an animal and the number of repeated desiccations that it had experienced (r_s_ = –0.23, P = 0.041, n = 79, Table 1). Thus, the frequency of mitosis tended to decline with the number of desiccation cycles and increase with number of storage cells. Since both mitosis occurrence and storage cell number tended to decline with number of repeated desiccations (see previous section), it is difficult to disentangle the causal relationships between mitosis, storage cell number and number of repeated desiccations.

**Table 1:** Proportion of specimens with mitotic storage cells and calculated mitotic index (no. mitotic cells/total number of cells per individual) for different desiccation cycle groups.

Number of desiccations	Proportion of specimens with mitotic storage cells	Mean mitotic index(SD)
0	0.32	0.21 (0.60)
1	0.40	0.57 (1.05)
2	0	0
3	0	0
4	0.20	1.16 (3.21)
5	0.20	0.12 (0.35)
6	0	0

The estimates of mitotic index include also individuals with no mitotic cells (mitotic index = 0). Controls (0 desiccations) include estimates from 19 specimens for proportion specimens with mitotic cells and 20 specimens for mitotic index, while the 1–6 desiccations include 10 specimens.

## Discussion

**Fig 5:**
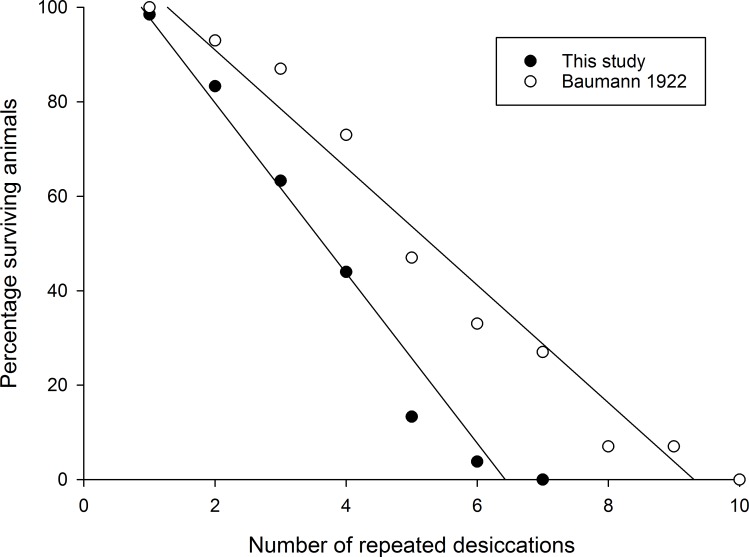
Percentage surviving tardigrades after repeated number of desiccations estimated in the current study and in that reported by Baumann [18]. Note that survival data from the current study were calculated based on estimated rates of survival for each desiccation cycle, not on the original number of animals, since 10 surviving animals were removed for analysis after each desiccation cycle. Initial number of animals: 400 in this study, 15 in Baumann´s study [18]. See main text for more detailed explanation.

Our study shows that tardigrades of the species *R*. *coronifer* are able to survive a maximum of 6 repeated desiccations under non-cultured conditions, with declining survival rates as the number of desiccations increased. This result is slightly lower than in the study by Baumann [18] who reported animals surviving up to 9 desiccation cycles. Fig 5 compares these two studies with respect to the proportion of the *initial population* of animals surviving after different numbers of desiccation cycles. Since we removed 10 specimens after each desiccation cycle, the proportion of survivals in relation to the initial population were calculated based on the specific desiccation cycle survival rate (given in Table A in S1 File) and the number of animals entering each cycle had we not removed those 10 surviving specimens. Both figures suggest that *R*. *coronifer* has a steeper decline in survival rate with increased number of desiccation cycles, compared to Baumann [18]. However, Baumann [18] only used 15 animals in total, and the last desiccation cycles included very few animals (the 9^th^ only one animal, which survived!). He also used a different desiccation-hydration schedule, with 24 hour desiccations (at 35–45% relative humidity), and 15 minute rehydrations. His results must therefore be taken with some caution. In contrast to Baumann [18] and the current study, Hengherr et al. [20] did not observe a decline in the probability to survive a cycle of anhydrobiosis over 9 consecutive desiccations (recovery rates were 88–100%), but the animals were allowed to feed and replenish their energy stores for 7 days between the desiccations, which naturally could have influenced the results.

The decline in the probability of a tardigrade to survive a desiccation cycle with number of previous desiccations suggests a progressive deterioration in the functions underlying anhydrobiosis. One plausible explanation for this limitation on multiple desiccations is that the animals eventually reach an energetic constraint, where the physiological processes necessary for the transition to and from the anhydrobiotic state is not supported by available energy. In support of this explanation our analyses showed a significant reduction in the number of storage cells after the fifth desiccation, indicating reabsorption of cells. Reabsorption of storage cells was also reported by Węglarska [25] as an effect of starvation in specimens of the eutardigrade *Dactylobiotus dispar* Murray, 1907 (formerly: *Macrobiotus dispar*). Howeverowever, data from another experiment in *R*. *coronifer* in which animals were starved for several days under continuously hydrated conditions at room temperature did not show a significant decline in number of storage cells (Czernekova & Jönsson, unpublished data). It is therefore not likely that the reduced storage cell number in this study was related to starvation during the short (5 hour) repeated periods of hydration.

Energy depletion connected to anhydrobiosis is also expected to lead to reduced size of the storage cells, as previously shown by Jönsson and Rebecchi [15]. In that study, storage cell area decreased by 14% over a single desiccation cycle in *R*. *coronifer*, corresponding to a decrease in cell volume by 22%. Decreased storage cell size has also been documented in the late phase of oogenesis, when energy investment into the eggs are high [15,26]. At this phase, food intake is prevented partly due to body cavity constraints, and partly because the feeding apparatus is expelled in connection with the onset of moulting (a stage called “simplex”). Reuner et al. [16] also reported significantly reduced storage cell size in three eutardigrade species (*M*. *tardigradum*, *Paramacrobiotus tonollii* and *Macrobiotus sapiens)* in response to 7 days of starvation, and in *M*. *tardigradum* in response to one cycle of anhydrobiosis. However, the number of storage cells in that study [16] was not affected neither by starvation nor by a period of anhydrobiosis. In our study, no significant reduction in cell size was found, even after several desiccation cycles. We also found no evidence that the energy depositions represented by gut content were depleted by repeated desiccation, a finding that was also reported by Baumann [18] in his study on repeated desiccation. Several previous studies on *R*. *coronifer* show that this species is able to survive continuous starvation at room temperature for many days and even weeks [27,28]. The declining survival rates after multiple desiccations therefore cannot be due to depletion of energy from the hydrated periods. The energy demands and role of storage cells in anhydrobiotic survival of *R*. *coronifer* therefore remains unclear.

The decline in survival with more desiccation cycles, particularly clear in the 5^th^ and 6^th^ cycle, corresponded with a higher proportion of animals that were unable to contract properly and create tuns when they were desiccated. This was also observed by Baumann [18], who suggested that the animals after a number (4 to 7) of repeated desiccations were unable to produce a cuticular secretion that prevented too rapid desiccation, resulting in uncontrolled contraction of the body and its organs and therefore incomplete tun formation. Our results on changes in body morphology over repeated desiccations are thus fully compatible with those of Baumann [18], but we did not evaluate any characteristics of the cuticle. Whether the failed tun formation after repeated desiccations is related to incomplete cuticular secretion remains to be studied, and ultrastructure analyses of cuticle and epidermis in animals exposed to different number of repeated desiccations would be highly interesting in this context.

The overall frequency of mitosis found in this study was similar to that reported in Czernekova and Jönsson [29], who found mitosis in 18.3% of the adult individuals of *R*. *coronifer*, and a mitotic index of 1.47% (based on individuals where mitotic cells were found). In that study it was also found that a higher frequency of mitosis was connected with the period of moulting which usually corresponds to the late phase of egg development. As mentioned above, that period is also characterized by smaller storage cells [15,26], but neither in the study by Czernekova and Jönsson [29] nor in the current study was cell size found to be associated with frequency of mitosis. Instead, the frequency of mitosis in storage cells tended to decline with the number of repeated desiccations and the total number of storage cells. Since the latter variable also declined with the number of repeated desiccations it is difficult to know if storage cell number directly affected mitosis frequency, or if both mitosis frequency and storage cell number were influenced by some other common factor related to repeated desiccation. However, the possibility that frequency of mitosis in storage cell is stimulated by energetic stress receives no support from our study, since this would predict an increase in mitosis with repeated desiccations, rather than the observed decrease.

In conclusion, our study clearly shows that the ability of *Richtersius coronifer* to enter and successfully leave the anhydrobiotic state declines with the number of previous anhydrobiotic cycles experienced, thus verifying a limit of how many times this tardigrade can enter anhydrobiosis in a row. The causal explanation behind this decline in anhydrobiotic performance is unclear, but increased inability to morphologically rearrange the body into a proper tun seems to be involved. The ultimate physiological or energetic reason for this remains to be documented.

### Compliance with Ethical Standards

Ethical approval: This chapter does not contain any studies with human participants performed by any of the authors.

## Supporting Information

S1 Fig a) Two desiccated specimens of *Richtersius coronifer* after proper tun formation. b) A desiccated specimens of *Richtersius coronifer* after semi-tun formation. c) A desiccated specimens of *Richtersius coronifer* in an extended (non-tun) state.
(JPG)

S2 Fig Mitotic storage cell of *Richtersius coronifer*. Scale bar: 10μm.
(TIF)

S1 File **Table A. Survival estimates for different number of repeated desiccation cycles, and the number and percentage of specimens that were in semi-tun or extended state.** ^a^ The total number of specimens were divided into 10 replicate samples. ^b^ Survival was evaluated after 3 and 5 hours of rehydration, except for specimens that were chosen for morphometry and mitosis analyses, which were evaluated 3 hours after rehydration. ^c^ Specimens with irregular tun were characterized as “semi-tun stage”. ^d^ Specimens that did not contract during desiccation. See Method section for more information. **Table B. Mean number and size (diameter) of storage cells after repeated cycles of desiccations.** Estimates for group 0 (controls) were based on 19–20 specimens, while estimates for the other groups were based on 10 specimens. Four outliers with cell diameters > 20 um were removed from the data; two from group 0 (5440 and 8662 um^3^), one from group 1 (4571 um^3^), and one from group 6 (10409 um^3^).
(DOC)
