# Supplementary figures and images for: Experimentally Induced Repeated Anhydrobiosis in the Eutardigrade Richtersius coronifer
Source: PLoS One. 2016 Nov 9;11(11):e0164062. doi: 10.1371/journal.pone.0164062 (PMC5102368; doi:10.1371/journal.pone.0164062)

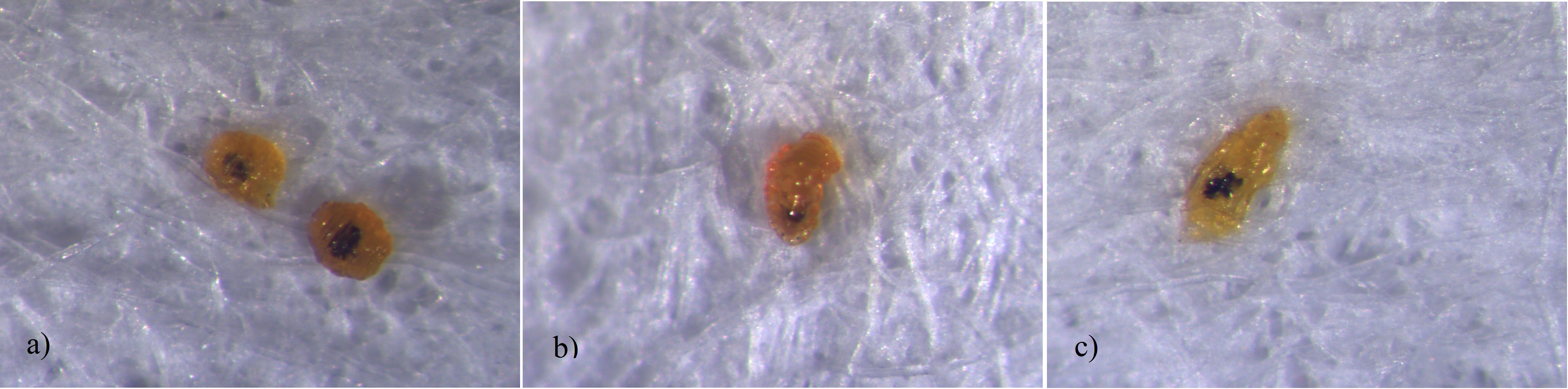

Supplement: S1 Fig — a) Two desiccated specimens of Richtersius coronifer after proper tun formation. b) A desiccated specimens of Richtersius coronifer after semi-tun formation. c) A desiccated specimens of Richtersius coronifer in an extended (non-tun) state. (JPG) [file pone.0164062.s001.jpg]

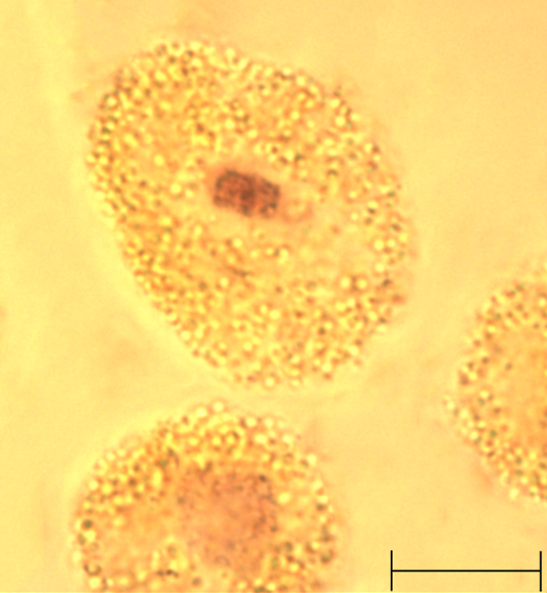

Supplement: S2 Fig — Scale bar: 10μm. (TIF) [file pone.0164062.s002.tif]
