# Supplementary material for: Experimentally Induced Repeated Anhydrobiosis in the Eutardigrade Richtersius coronifer
Source: PLoS One. 2016 Nov 9;11(11):e0164062. doi: 10.1371/journal.pone.0164062 (PMC5102368; doi:10.1371/journal.pone.0164062)
Supplement: S1 File — Table A. Survival estimates for different number of repeated desiccation cycles, and the number and percentage of specimens that were in semi-tun or extended state. a The total number of specimens were divided into 10 replicate samples. b Survival was evaluated after 3 and 5 hours of rehydration, except for specimens that were chosen for morphometry and mitosis analyses, which were evaluated 3 hours after rehydration. c Specimens with irregular tun were characterized as “semi-tun stage”. d Specimens that did not contract during desiccation. See Method section for more information. Table B. Mean number and size (diameter) of storage cells after repeated cycles of desiccations. Estimates for group 0 (controls) were based on 19–20 specimens, while estimates for the other groups were based on 10 specimens. Four outliers with cell diameters > 20 um were removed from the data; two from group 0 (5440 and 8662 um3), one from group 1 (4571 um3), and one from group 6 (10409 um3). (DOC) [file pone.0164062.s003.doc]

**Supporting Information S1**

**Table A.** **Survival estimates for different number of repeated desiccation cycles, and the number and percentage of specimens that were in semi-tun or extended state.**

| Number of dehydration cycles | Specimens in dehydration^a^ cycle | Mean (SD) survival^b^ (%) | Mean (SD) proportion semi-tun specimens^c^ (%) | Mean (SD) proportion extended specimens^d^ (%) | Mean (SD) proportion with dark gut content (%) |
| --- | --- | --- | --- | --- | --- |
| 1 | 400 | 98.5 (2.0) | 5.3 (3.6) | 0 (-) | 65.0 (8.0) |
| 2 | 384 | 84.6 (8.1) | 14.3 (8.3) | 7.0 (7.0) | 67.7 (8.0) |
| 3 | 315 | 75.9 (10.5) | 16.2 (10.6) | 7.4 (6.5) | 72.6 (8.2) |
| 4 | 229 | 69.4 (9.6) | 25.0 (12.3) | 10.0 (22.9) | 76.0 (6.3) |
| 5 | 149 | 30.2 (11.7) | 54.3 (16.3) | 17.4 (9.5) | 75.9 (7.4) |
| 6 | 35 | 28.6 (37.9) | 41.7 (29.8) | 15.8 (26.5) | 82.5 (19.5) |

^a^ The total number of specimens were divided into 10 replicate samples. ^b^ Survival was evaluated after 5 hours of rehydration, except for specimens that were chosen for morphometry and mitosis analyses, which were evaluated 3 hours after rehydration. ^c^ Specimens with irregular tun were characterized as “semi-tun stage”. ^d^ Specimens that did not contract during desiccation. See Method section for more information.

**Table B.** **Mean number and size (diameter) of storage cells after repeated cycles of desiccations.**

| Number of desiccations |  | Mean body length (SD) (um) | Mean number (SD) of storage cells | Mean volume (SD) of storage cells (um^3^) |
| --- | --- | --- | --- | --- |
| 0 |  | 696.1 (68.2) | 844 (214) | 1099 (543) |
| 1 |  | 621.7 (124.5) | 681 (146) | 1315 (708) |
| 2 |  | 690.5 (79.3) | 793 (225) | 1265 (425) |
| 3 |  | 645.3 (95.1) | 725 (213) | 1265 (506) |
| 4 |  | 606.0 (76.3) | 781 (200) | 1231 (440) |
| 5 |  | 631.9 (43.8) | 579 (210) | 1554 (670) |
| 6 |  | 656.1 (93.9) | 547 (116) | 1500 (526) |

Estimates for group 0 (controls) were based on 19-20 specimens, while estimates for the other groups were based on 10 specimens. Four outliers with cell diameters > 20 um were removed from the data; two from group 0 (5440 and 8662 um^3^), one from group 1 (4571 um^3^), and one from group 6 (10409 um^3^).
